# Neuroanatomical and Microglial Alterations in the Striatum of Levodopa-Treated, Dyskinetic Hemi-Parkinsonian Rats

**DOI:** 10.3389/fnins.2020.567222

**Published:** 2020-09-15

**Authors:** Edward J. R. Fletcher, Clare J. Finlay, Ana Amor Lopez, William R. Crum, Anthony C. Vernon, Susan Duty

**Affiliations:** ^1^Wolfson Centre for Age Related Diseases, Wolfson Wing, Institute of Psychiatry, Psychology and Neuroscience, King’s College London, London, United Kingdom; ^2^Department of Neuroimaging, Institute of Psychiatry, Psychology and Neuroscience, King’s College London, London, United Kingdom; ^3^Department of Basic and Clinical Neuroscience, Maurice Wohl Clinical Neuroscience Institute, Institute of Psychiatry, Psychology and Neuroscience, King’s College London, London, United Kingdom; ^4^MRC Centre for Neurodevelopmental Disorders, King’s College London, London, United Kingdom

**Keywords:** abnormal involuntary movements, astrocytes, 6-Hydroxydopamine, levodopa-Induced dyskinesia, magnetic resonance imaging, microglia, microvasculature

## Abstract

Dyskinesia associated with chronic levodopa treatment in Parkinson’s disease is associated with maladaptive striatal plasticity. The objective of this study was to examine whether macroscale structural changes, as captured by magnetic resonance imaging (MRI) accompany this plasticity and to identify plausible cellular contributors in a rodent model of levodopa-induced dyskinesia. Adult male Sprague-Dawley rats were rendered hemi-parkinsonian by stereotaxic injection of 6-hydroxydopamine into the left medial forebrain bundle prior to chronic treatment with saline (control) or levodopa to induce abnormal involuntary movements (AIMs), reflective of dyskinesia. Perfusion-fixed brains underwent *ex vivo* structural MRI before sectioning and staining for cellular markers. Chronic treatment with levodopa induced significant AIMs (*p* < 0.0001 versus saline). The absolute volume of the ipsilateral, lesioned striatum was increased in levodopa-treated rats resulting in a significant difference in percentage volume change when compared to saline-treated rats (*p* < 0.01). Moreover, a significant positive correlation was found between this volume change and AIMs scores for individual levodopa-treated rats (*r* = 0.96; *p* < 0.01). The density of Iba1+ cells was increased within the lesioned versus intact striatum (*p* < 0.01) with no difference between treatment groups. Conversely, Iba1+ microglia soma size was significantly increased (*p* < 0.01) in the lesioned striatum of levodopa-treated but not saline-treated rats. Soma size was not, however, significantly correlated with either AIMs or MRI volume change. Although GFAP+ astrocytes were elevated in the lesioned versus intact striatum (*p* < 0.001), there was no difference between treatment groups. No statistically significant effects of either lesion or treatment on RECA1, a marker for blood vessels, were observed. Collectively, these data suggest chronic levodopa treatment in 6-hydroxydopamine lesioned rats is associated with increased striatal volume that correlates with the development of AIMs. The accompanying increase in number and size of microglia, however, cannot alone explain this volume expansion. Further multi-modal studies are warranted to establish the brain-wide effects of chronic levodopa treatment.

## Introduction

Levodopa (L-DOPA) remains a first-line treatment for motor symptoms in many individuals affected by Parkinson’s disease (PD) (Schapira et al., 2009). Prolonged treatment, however, is associated with a decline in therapeutic efficacy and the development of debilitating L-DOPA-induced dyskinesia (LID) (Iravani and Jenner, 2011). This severely limits the long-term clinical utility of L-DOPA to treat PD motor symptoms and is associated with a significant negative impact on patient quality of life. Research efforts to unravel the neural correlates of LID in the clinic and in relevant animal models for LID are thus critical to address the gaps in our knowledge of LID pathogenesis. In this context, regions of the basal ganglia that regulate motor function and are dysfunctional in PD have been explored extensively in both dyskinetic PD patients and rodent LID models using functional magnetic resonance imaging (fMRI) and positron emission tomography (PET). Collectively, such studies provide evidence for both brain network and metabolic changes relating to the pathophysiology of LID (Brooks et al., 2000; Kuriakose and Stoessl, 2010; Cerasa et al., 2012, 2015; Herz et al., 2015, 2016; Berman et al., 2016). Specifically, L-DOPA treatment is associated with increased vascular perfusion (indexed by cerebral blood flow or [^15^O]-H_2_O PET) that is dissociated from metabolic changes (indexed by cerebral glucose utilization via [^18^F]-fluorodeoxyglucose PET) in the basal ganglia, motor and pre-frontal cortices (Hirano et al., 2008). Back translating these measures to rodent models for LID has resulted in similar observations (Ohlin et al., 2012; Lerner et al., 2016), paving the way to probing the underling physiological and cellular correlates of these functional changes. Evidence from such invasive studies strongly suggests that development of LID is associated with maladaptive synaptic plasticity in striatal medium spiny and cortical pyramidal neurons, with corresponding morphological changes such as increased dendritic spine density (Deutch et al., 2007; Zhang et al., 2013; Finlay et al., 2014; Nishijima et al., 2014; Suarez et al., 2014; Ueno et al., 2014, 2017).

Whether these cellular adaptations to L-DOPA treatment are accompanied by macro-structural changes in brain volume following LID is not known. This is relevant, since structural MRI changes are present in *de novo* PD patients and progress with increasing disease duration (Mak et al., 2015, 2017). Whether chronic exposure to L-DOPA modifies these changes, remains unclear. If true, this could have implications for interpretation of structural MRI data in medicated PD patients, particularly those with dyskinesia. In support of this view, structural MRI studies comparing dyskinetic and non-dyskinetic PD patients provide evidence that independent of the age of PD onset, dyskinetic PD patients are characterized by increased gray matter volume and thickness in the inferior frontal cortex, with additional region-specific changes depending on the age of onset of PD symptoms (Cerasa et al., 2011, 2013a,b). On the other hand, longitudinal structural MRI studies of PD patients have reported no relationship between changes in cortical thickness or volume and daily L-DOPA equivalents, although none of these patients were reported to be dyskinetic (Mak et al., 2017).

Separating medication from disease effects remains challenging in human imaging studies. Hence, whether anatomical changes in dyskinetic PD patients are a cause or consequence of L-DOPA treatment remains debated (Aron and Obeso, 2012; Vernon and Modo, 2012) and their precise cellular correlates have yet to be mapped. The combination of MRI (clinically comparable technology) with existing rodent models for LID (Lundblad et al., 2002; Duty and Jenner, 2011) offers a means to address these questions directly and no such studies exist in the literature. Therefore, in the current study, we build on our prior structural MRI work in 6-hydroxydopamine (6-OHDA) lesioned rats (Westphal et al., 2016) to determine whether chronic L-DOPA treatment is associated with macroscale changes in rat brain structure, detectable using MRI. In this study, we focussed our investigations *a priori* on the striatum based on the following rationale. First, a wealth of evidence from functional neuroimaging, electrophysiological and neuropathological studies implicates the striatum as central to the pathophysiology of LID in rodents (Ohlin et al., 2012; Calabresi et al., 2015; Fieblinger and Cenci, 2015). Second, previous work from our group has identified that the volume of the striatum in the ipsilateral (lesioned) hemisphere is statistically significantly reduced in two different rodent neurotoxin-based models for parkinsonism (Vernon et al., 2010, 2011; Westphal et al., 2016). This finding translates across species, since bilateral reductions in striatal volume are also found in primate neurotoxin-based models for parkinsonism (Modo et al., 2017). Moreover, this measure has clinical relevance, since longitudinal structural MRI studies provide evidence for a progressive decrease in the gray matter volume of the caudate nucleus and putamen of individuals with PD relative to healthy controls, which appears to be more prominent in the early stages of the disease (Lewis et al., 2016). Notably, the effect of L-DOPA treatment *per se* on striatal volume is also unknown. Finally, we aimed to identify some potential cellular correlates of any changes observed in striatal volume through focussed *post-mortem* investigations.

## Materials and Methods

### Animals

Adult male Sprague-Dawley rats (270–300g, Envigo, United Kingdom) were maintained in a temperature- and humidity-controlled environment with access to food and water *ad libitum*. All animal procedures adhere to the ARRIVE guidelines for pre-clinical animal studies, are in accordance with the United Kingdom Animals (Scientific Procedures) Act, 1986 and European Union Directive 2010/63/EU, and were approved by King’s College London Animal Welfare and Ethical Review Body. The experimenter was blinded to the treatment (saline or L-DOPA) for all behavioral and histological procedures. A total of 20 rats were used.

### Unilateral 6-OHDA Lesioning and Apomorphine-Induced Rotations

**FIGURE 1:**
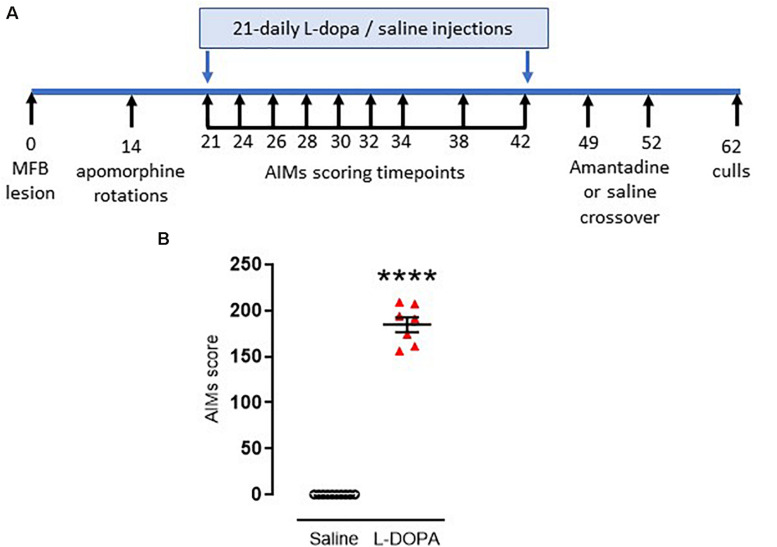
Timeline and confirmation for induction of dyskinesia in rats. **(A)** Timeline for induction and assessment of L-DOPA-induced abnormal involuntary movements (AIMs), reflective of dyskinesia, in rats subject to a 6-hydroxydopamine-lesion of the median forebrain bundle (MFB) on day 0. Full description is contained within the manuscript text. **(B)** Graph displaying individual AIMs scores obtained on the final day of priming with either L-DOPA (6.25 mg/kg s.c. with benserazide [15 mg/kg s.c.]; *n* = 7) or saline (*n* = 10). Horizontal bars indicate mean ± SEM. ****p < 0.0001 (Mann–Whitney *U* test) versus saline controls.

The *in vivo* study timeline is show in Figure 1A. At day 0, under isofluorane anesthesia (5% induction, 2–3% maintenance), all 20 rats were infused (0.5 μl/min) with 12.5 μg 6-hydroxydopamine (6-OHDA.HCl, Sigma-Aldrich) in 2.5 μl saline containing 0.2% ascorbic acid, into the left medial forebrain bundle (AP-2.6 mm, ML + 2.0 mm and DV-8.8 mm relative to bregma). Two weeks post-lesion, the full extent of the 6-OHDA lesion was confirmed using apomorphine-induced rotation (Duty and Jenner, 2011). Briefly, after 30 min acclimatization in rotometers, rats were injected with apomorphine (0.5 mg/kg, s.c.) and the net contraversive rotations were recorded over 90 min using Rotorat software (MedAssociates). This revealed 19 of the 20 rats to be fully lesioned (displayed 382 ± 32 net contraversive rotations over 90 min) and therefore valid for inclusion in the subsequent study.

### L-DOPA Treatment and Scoring of Abnormal Involuntary Movements

On day 21 post-lesion, dyskinesia priming commenced. Rats were injected daily for 21 days with either a dyskinesia-inducing dose of L-DOPA (e.g., Lundblad et al., 2002) (6.25 mg/kg s.c. with benserazide [15 mg/kg s.c.]; *n* = 9) or saline (1 ml/kg, s.c; *n* = 10). During the priming period, axial, limb and orolingual (ALO) abnormal involuntary movements (AIMs) were assessed at intervals (shown in Figure 1A) by an operator blinded to treatment, as an index of dyskinesia. Briefly, rats were placed in a clear acrylic cylinder (diameter 40 cm × height 30 cm) for 30 min acclimatization before injection of L-DOPA or saline. Rats were then scored for 1 min every 20 min over a 180 min period. Scoring was performed using established criteria. For severity of each of the ALO AIMs (Cenci et al., 1998): 0, absent; 1, occasional (<50% time); 2, frequent (>50% time); 3, continuous but can be interrupted by sensory stimuli; 4, continuous and cannot be interrupted by sensory stimuli (Winkler et al., 2002). For amplitude, axial and limb scores of 1–4 were assigned exactly as previously described (Winkler et al., 2002) with additional scores for orolingual amplitude of: 1, vacuous chewing; 2, tongue protrusion.

The maximum possible score for an animal was 360: 144 for each of the axial and forelimb subsets (4 for amplitude × 4 for severity × 9 time points = 144) and a further 72 for the orolingual subset (2 for amplitude × 4 for severity × 9 time points = 72). The total AIMs score calculated on the final day of L-DOPA priming (day 42 post-lesion) was used to assess the degree of dyskinesia established for each animal. In line with previous studies (Rangel-Barajas et al., 2011; Albarran-Bravo et al., 2019), only L-DOPA-treated animals showing severe dyskinesia, defined as scoring >0.3× maximum possible score (>108 in this case) were selected for further study. Seven of the nine L-DOPA-treated rats met this threshold for classification as severely dyskinetic.

One week later, the ability of the anti-dyskinetic drug amantadine to reverse the established AIMs was assessed. Briefly, all rats were injected with amantadine HCl (40 mg/kg, s.c.) or saline (1 ml/kg, s.c.) in a randomized and blinded cross-over design with 72h between doses (hence dosed on days 49 and 52 post lesion [Figure 1]). Thirty minutes post amantadine or saline, rats were administered L-DOPA (6.25 mg/kg s.c. with benserazide [15 mg/kg s.c.] and AIMs were assessed for one minute every 20 min over a 180 min period.

### Brain Processing for MRI Assessments

Ten days after the last L-DOPA/saline challenge (day 62), rats were terminally anesthetized with sodium pentobarbital (250 mg, i.p.), transcardially perfused with saline and subsequently perfused with 4% paraformaldehyde (PFA). Rat heads were removed and, with the brain left *in situ*, were placed in PFA. Twenty-four hours later the heads were washed in PBS/0.05% sodium azide at 4°C for 3 weeks prior to imaging to allow rehydration of the tissue. We chose to collect *ex vivo* data for the pragmatic reason that the animal facility and MR imaging suite were not co-located on the same physical site and to provide superior resolution. There are, however, important trade-offs to consider between *in vivo* and *ex vivo* imaging (Lerch et al., 2012; Ma et al., 2019). In particular, sample preparation (perfusion and tissue fixation) for *ex vivo* imaging may cause morphological disruption to the tissues, which could affect interpretation of the data (Ma et al., 2019). Total brain volume and some regional gray matter structures also shrinks post-perfusion (Vernon et al., 2011; Holmes et al., 2017; Ma et al., 2019). Prior work, however, including our own, suggests that the majority of group-level differences in regional volume are preserved and were confirmed post-mortem using stereology (Vernon et al., 2011; Ma et al., 2019) The post-mortem brain processing method used, as well as the use of different RF coils, scanning sequences and improper attention to gradient calibration are, however, additional sources of variance that can contribute to systematic differences between *in vivo* and *ex vivo* MR data, precluding a definitive conclusion (Ma et al., 2019). Taken together, the choice of *ex vivo* MRI is consistent with the aims of this study, but longitudinal *in vivo* studies to confirm and extend our initial *ex vivo* findings are an important future goal.

### Magnetic Resonance Imaging

A 7T small-bore horizontal magnet MRI scanner (Agilent Technologies Inc., Santa Clara, CA, United States) equipped with a custom-made quadrature volume radiofrequency coil (43 mm inner diameter, Magnetic Resonance Laboratory, Oxford) was used for all *post-mortem* magnetic resonance acquisition. Anatomical MRI were acquired using a Fast Spin Echo sequence: repetition time/effective echo time = 4000/60 ms; averages = 8; field of view = 30 × 30 mm; matrix size = 128 × 128 mm; 45 contiguous coronal slices, 0.6 mm thick giving an in-plane resolution of 125 μm. Scans were acquired blinded to treatment status in a random order, interspersed with phantoms to ensure consistent operation of the magnet. The magnetic resonance images were converted offline to NIFTI file format and visually inspected for motion or intensity artifacts. No scans were excluded on this basis.

### Volumetric Segmentation of the Rat Striatum

Absolute volumes of the striatum (mm^3^) were derived from the *ex vivo* MR images by two reviewers (AAL and ACV), on a slice-by-slice basis in the coronal plane using the polygon contour tool in ITK-snap (Yushkevich et al., 2006), blinded to treatment group. The region of interest (ROI) contours were traced in the left and right brain hemispheres at low magnification followed by manual correction of borders at higher magnification based on previously published criteria from our group (Vernon et al., 2011; Vernon and Modo, 2012). As described in the Introduction, the striatum was selected *a priori* based on previous findings implicating this region in LID (Ohlin et al., 2012; Calabresi et al., 2015; Fieblinger and Cenci, 2015). We additionally tested for asymmetry in the volumes of the striatum using an asymmetry index (AI) defined as (L-R)/(L + R), where L and R are the left and right measures and in which positive and negative AS values indicate leftward and rightward asymmetry, respectively (Iturria-Medina et al., 2011; Ratnarajah et al., 2013). An illustration of the contours used to segment the rat striatum is shown in Supplementary File 1A.

### Immunohistochemistry

Following *ex vivo* MRI, brains were removed from the skulls and submerged in 30% sucrose/PBS with 0.05% sodium azide for 2 days before embedding in 10% porcine gelatin. Blocked brains were then post-fixed in PFA for 24 h before immersing in 30% sucrose/PBS with 0.05% sodium azide. The operator was blinded to treatment during subsequent immunohistochemistry and data analysis. Twelve series of rostral to caudal 40 μm thick coronal sections were cut through the striatum using a freezing sledge microtome and stored free-floating in PBS with 0.05% sodium azide. One series (nine sections spanning the entire rostrocaudal axis; AP + 1.7 – AP-2.5) were used per animal for each of the immunohistochemistry protocols. Sections were incubated for 10 min with 3% H_2_O_2_ and 10% methanol in dH_2_O, washed thrice in TBS then incubated for 60 min in 0.2% triton-X100 in TBS containing 3% normal goat serum (NGS) or, in the case of anti-Iba-1, 1% bovine serum albumin (BSA). Sections were incubated overnight in rabbit polyclonal anti-GFAP (glial fibrillary acidic protein; 1:10,000; ab7260), goat polyclonal anti-Iba1 (Ionized calcium binding adaptor molecule 1; 1:2000; ab5076) or mouse polyclonal anti-RECA1 (rat endothelial cell antigen-1; 1:2000; ab9774) at room temperature. Sections were then washed in TBS before incubation in 1% BSA or 3% NGS, as before, containing the appropriate biotinylated secondary antibody: goat anti-rabbit (1:1000; BA-1000), horse anti-goat (1:1000; BA-9500) or horse anti-mouse (1:1000; BA-2001) for 1 h at room temperature. Sections were then incubated in streptavidin horseradish peroxidise binding complex (Vector Labs; SA-5004) for 30 min before a final TBS wash and immersion in developing solution (0.05% 3,3′diaminobenzidine in TBS with 0.01% H_2_O_2_) for 10 min. Sections were mounted onto slides, left to dry overnight then dehydrated before being cover-slipped.

### Digital Image Acquisition & Image Analysis

A Zeiss brightfield microscope (20x objective) was used to capture panoramic TIFF images of the whole striatum from each of nine sections per animal (Axiovision 4.6). TIFF files were converted to an 8-bit/binary image before analysis using FIJI software (National Institutes of Health). Using the Freehand tool in FIJI, both the entire ipsilateral intact and contralateral lesioned striata were outlined with the aid of The Rat Brain Atlas (Paxinos and Watson, 2007). Image analysis was then performed as described below for each marker, across all nine sections per animal, spanning the entire rostrocaudal axis; AP + 1.7 – AP-2.5).

The RECA1+ vascular staining was analyzed for the number of microvessels (i.e., stained vasculature in phase with the 40 μm section) using the Skeletal analysis tool in FIJI. Briefly, the 8-bit/binary image was skeletonized then skeletal analysis was conducted (shortest branch prune cycle method). To eliminate false readings, all microvessels below 20 μm in length were removed as these could not be confidently identified as vasculature. For analysis of GFAP+ astrocyte staining, mean gray value (MGV) measurements taken. For analysis of Iba1+ microglia, both the number and soma size were quantified. Thresholding was set to a range that captured the entire cell fully without introducing noise or outliers. This range was kept consistent throughout the analysis with size threshold limits set to 25–150 μm^2^. Cell soma sizes and number of microglia were then determined by the Analyze Particles tool with cell density calculated per mm^2^ of striatum. For each parameter, quantification was averaged across all nine sections of intact or lesion striatum per animal, with final group means obtained per treatment.

### Microglial Morphometric Analysis

A more detailed analysis of microglial morphometry was conducted using Iba1 fractal analysis, as previously described (Young and Morrison, 2018). In brief, using the image analysis program FIJI (available at https://imagej.net/Fiji), the rectangle tool was used to select a region of interest (ROI) that captured a single randomly selected Iba1+ cell. The rectangle size was kept constant for all other cell ROIs within the dataset (in this instance 89.82 × 89.82 μm, 277 × 277 pixels). For each animal, 3 cells per striatal hemisphere per section were analyzed, resulting in 27 randomly selected microglia from each striata per rat. ROIs were converted to 8-bit and then greyscale before the FFT bandpass filter was applied. The cell was then transformed into a binary black and white image and any outliers removed with the despeckle tool. Manual removal of pixels from other neighboring cells and/or the addition of pixels to join broken processes from the isolated cell was conducted with the paintbrush tool (the original image of the cell was used as a template to ensure the correct deletion/addition of pixels). The final ROI was then saved and analyzed using the FracLac plugin (Karperien et al., 2013). Under the FracLac BC (box counting) option, the Num G value (Grid Design setting) was set to 4 and the metric box ticked for Graphics Options to allow hull and circle measurements. Once set, these parameters were used for the scan of each cell image.

Six metrics were measured to analyze microglial morphometry. All six were calculated using the FracLac plugin for FIJI:

#### Hull Area and Perimeter

The hull, or convex hull, is formed by a series of connected straight lines that enclose all of the pixels of the cell. The hull is therefore the smallest polygon that can capture the entire cell. The area and perimeter of this hull was measured to assess the overall size of the microglia.

#### Hull Circularity

This is a measure of likeness to a circle, where a perfect circle would be 1. This indicates the degree of microglial activation. Hull circularity = (4πHull area)/(Hull perimeter)^2^.

#### Lacunarity

This is a measure of heterogeneity and rotational variance that reflects gap structure. A higher lacunarity implies a cell possesses increased heterogeneity and thus differently sized spaces or gaps within the shape. A low lacunarity suggests a structure that has increased homogeneity where the structure has regularly spaced and sized gaps. In this instance microglia with more complex branching will give rise to more irregular gaps resulting in a higher lacunarity value.

#### Fractal Dimension

This is a measure of shape complexity, or more precisely the alteration of detail in reference to a change of scale. FracLac utilizes box counting sampling to capture the foreground pixels of the cell with increasingly smaller grid pattern calibes. The box count data and box calibe size can then be plotted via logarithmic regression to calculate a slope, the exponent of which provides the fractal dimension value. Box counting is sensitive to the morphological aspects of microglia such as the degree of branching and cellular shape (Karperien et al., 2013), therefore it can indicate levels of ramification in a microglial dataset.

#### Density

Density is calculated as the cell area (μm^2^) divided by the total hull area (μm^2^) and reflects the density of each cell so is quite distinct from the striatal density measurement described above.

### Statistical Analyses

All statistical analyses were performed using Prism (version 7; Graph Pad Software Inc., La Jolla, CA, United States). Group differences in overall AIMs scores were compared using a Mann–Whitney *U* test. Data obtained from the amantadine experiment were analyzed with a two-tailed paired *t*-test. For MRI manual segmentation data, in order to compensate for possible differences between animals and compensatory effects due to the unilateral nature of the lesion model, volume data from the left, ipsilateral, lesioned hemisphere were expressed a percentage change relative to the right, contralateral, intact hemisphere in each animal. To control for the possibility that our normalization approach may bias the data, we also calculated an asymmetry index (AI) using the raw absolute volumes for each hemisphere, as defined in the MRI methods. Comparisons between treatment groups (saline or L-DOPA) for each measure (% volume change and AI) were performed using a two-tailed *t*-test. For all *post-mortem* immunohistochemical comparisons, main effects of hemisphere (ipsilateral, contralateral) or treatment (saline, L-DOPA) and their interaction were assessed using two-way ANOVA. Where appropriate, *post hoc* tests were conducted using Tukey’s test. Comparison between treatment groups (saline or L-DOPA) for % changes in soma size were performed using a two-tailed *t*-test. Correlations between selected parameters were performed for the L-DOPA treatment group using bivariate Pearson’s correlation test. Statistical significance was set at α = 0.05.

## Results

### L-DOPA Treatment in the 6-OHDA-Lesioned Rat Induces Abnormal Involuntary Movements That Are Sensitive to Amantadine

As noted in the Methods, 19 of the 20 rats were successfully lesioned with 6-OHDA, displaying 382 ± 32 (mean ± S.E.M.) net contraversive rotations in 90 min when challenged acutely with apomorphine (0.5 mg/kg, s.c.). These were randomly allocated to saline (control) or L-DOPA treatment groups. Following 21 days priming with L-DOPA (6.25 mg/kg) plus benserazide (15 mg/kg) 77% (7/9) 6-OHDA-lesioned rats developed AIMs scores > 108, classifying them as severely dyskinetic (Figure 1B). The two remaining animals with AIMs scores of 57 and 94 were not selected for further study. As expected, none of the 6-OHDA-lesioned rats treated with saline (*n* = 10) expressed any AIMs (*p* < 0.0001 versus L-DOPA). An acute amantadine challenge (40 mg/kg) significantly reduced the total AIMs in L-DOPA-treated rats from 191 ± 8 to 133 ± 16 (*t* = 6.; df = 6; *p* < 0.001). These data confirm this as a valid model of LID in rodents that has face, construct and predictive validity (Lundblad et al., 2002; Dekundy et al., 2007; Duty and Jenner, 2011).

### Striatal Volume Is Increased in 6-OHDA Lesioned Rats Treated With L-DOPA as Compared to Saline

**FIGURE 2:**
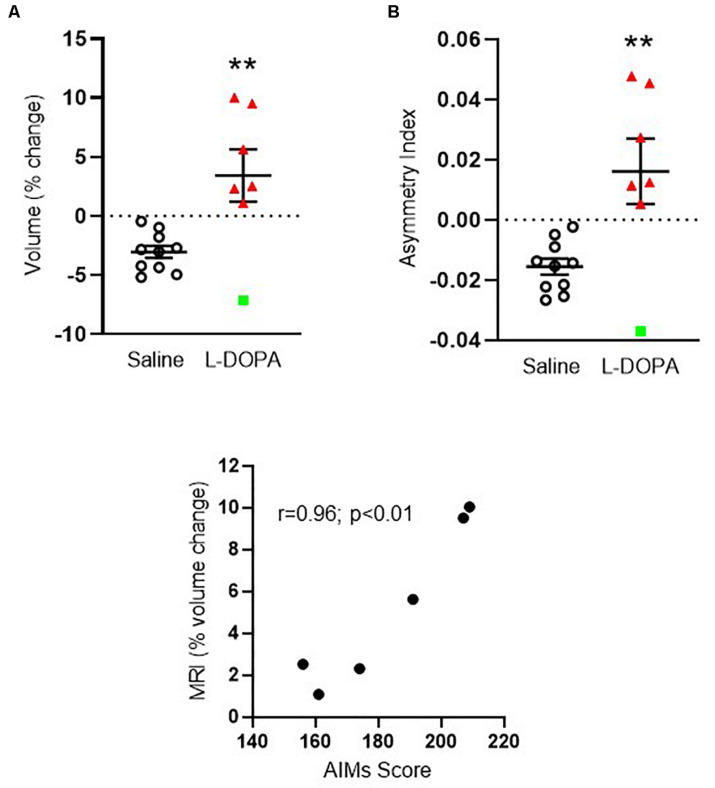
Apparent volume changes in the striatum of 6-OHDA-lesioned rats treated for 21 days with either L-DOPA (dyskinetic) or saline (control). **(A)** Chronic L-DOPA increases the absolute volume (mm^3^) of the striatum in 6-OHDA lesioned animals displaying severe dyskinesia (*n* = 7) compared to 6-OHDA lesioned animals treated with saline (*n* = 10). Data from the ipsilateral, lesioned striatum are expressed as a percentage change relative to the contralateral, intact striatum. **p < 0.01 (two-tailed *t*-test). **(B)** Asymmetry index depicting alterations in striatal volume. A positive score suggests leftward asymmetry, consistent with an increase in the volume of the ipsilateral, lesioned striatum. **p < 0.01; (two-tailed *t*-test). Error bars indicate mean ± SEM. Green square indicates an outlier in MRI analysis which is shown for transparency, but not included in the statistical analysis. **(C)** Scatter plot showing positive correlation between the percentage striatal volume change and AIMs scores for individual rats, (excluding MRI outlier), in the L-DOPA-treatment group (*n* = 6; *p* < 0.01; Bivariate Pearson’s correlation).

We first compared the volume of the ipsilateral (left, lesioned) striatum, expressed as a percentage of the contralateral (right, intact) hemisphere in each animal between treatment groups. Only animals displaying severe dyskinesia were included from the L-DOPA group. Hence, *n* = 10 6-OHDA lesioned rats treated with saline were compared to *n* = 7 6-OHDA lesioned rats treated with L-DOPA. A mean negative percentage change in striatal volume was observed in saline-treated rats of ([mean ± SEM] -3.05 ± 0.52), consistent with shrinkage of the ispilateral striatum relative to the intact, contralateral hemisphere and in line with our previous MRI findings in this model (Westphal et al., 2016). In contrast, a mean positive percentage change was observed in L-DOPA treated rats (+ 3.42 ± 2.2) suggestive of a volume increase in the ipsilateral hemisphere. Direct comparison of these values reached statistical significance (*t* = 3.36; df = 15; *p* < 0.01) with an effect size (Cohen’s *d*) of 1.5 (Figure 2A). To confirm this finding, we calculated the asymmetry index (AI) using the raw absolute volumes (mm^3^) of the striatum in each hemisphere, for each animal, in each treatment group. This revealed a statistically significant positive AI score (*t* = 3.32; df = 15; *p* < 0.01) with an identical effect size (Cohen’s *d*) of 1.5 (Figure 2B). These data are consistent with leftward asymmetry (i.e., greater volume in the left, ipsilateral lesioned hemisphere, as compared to the right, contralateral intact hemisphere). Of note, the volume change of one L-DOPA treated rat (depicted by green square symbol in Figures 2A,B) fell > 2 standard deviations away from the group mean. Hence, this animal was excluded from the statistical analysis for correlations performed against MRI percentage volume change. There was a significant positive correlation between the percentage volume change and AIMs scores for individual rats in the L-DOPA-treatment group (*r* = 0.96; *p* < 0.01; *n* = 6; Figure 2C).

### 6-OHDA-Lesioned Rats Treated With L-DOPA Have Increased Density of Enlarged Microglia Within Their Lesioned Striatum Compared to Saline Controls

**FIGURE 3:**
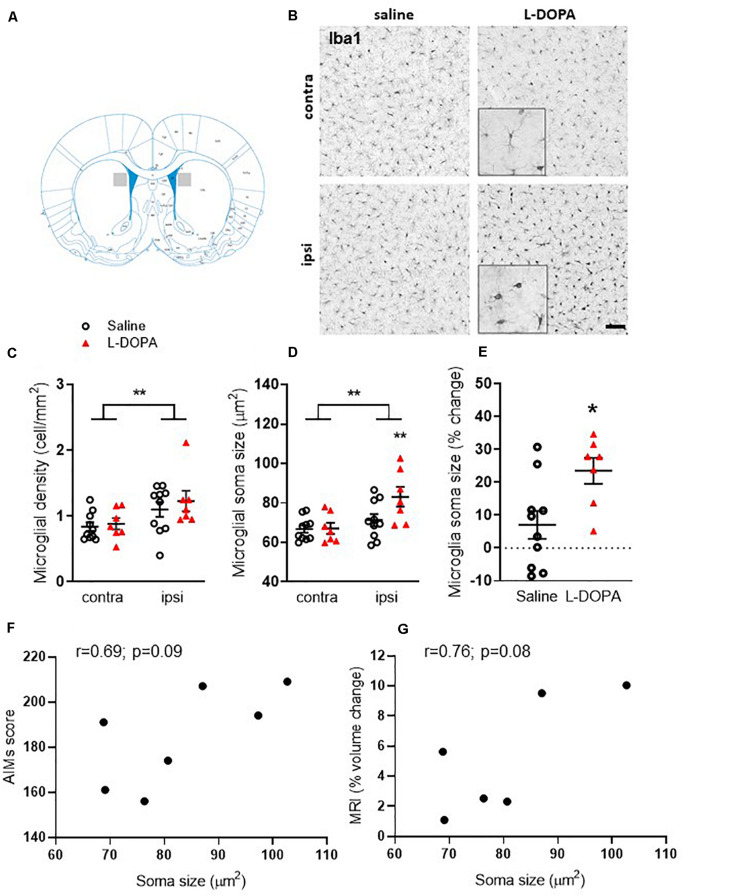
Assessment of striatal microglia density and soma size in L-DOPA-treated (dyskinetic) versus saline-treated (control) rats. Figures represent **(A)**. Schematic with shaded area to illustrate striatal region represented in low power images presented in B **(B)** Representative photomicrographs of Iba1+ microglia within the contralateral (contra) intact and ipsilateral (ipsi) lesioned striatum of saline- and L-DOPA -treated rats. Scale bar = 100 μm. Insert shows magnified 100 μm square. **(C)** Density (cells/mm^2^) and **(D)** soma size of microglia within the striatum. ** above horizontal bar denote an overall effect of lesion (*p* < 0.01) while ** below bar denotes *p* < 0.01 versus contralateral hemisphere in same treatment group (two-way ANOVA with Tukey *post hoc* test). **(E)** L-DOPA treatment increases microglia soma size significantly more than saline. Data from the ipsilateral, lesioned striatum are expressed as a percentage change relative to the contralateral, intact striatum of each treatment group. *p < 0.05 (two-tailed *t*-test). Bars show mean ± SEM. Saline-treated: *n* = 10; L-DOPA -treated: *n* = 7. **(F,G)** Scatter plots showing correlation between microglial soma size and AIMs score (*n* = 7) or percentage volume change (*n* = 6) for individual rats in the L-DOPA-treatment group (*p* > 0.05; Bivariate Pearson’s correlation).

In order to establish plausible cellular correlates of the observed volume increase in the ipsilateral lesioned striatum of L-DOPA treated 6-OHDA lesioned rats, we next focussed on *post-mortem* investigations. Microgliosis has previously been suggested to contribute to the development of AIMs (Bortolanza et al., 2015; Pisanu et al., 2018) therefore we analyzed microglial cell density and soma size. Iba1+ microglia were significantly higher in density (cells/mm^2^) within the lesioned striatum compared to the intact for both the saline and L-DOPA treatment groups (*F*_1_,_30_ = 8, *p* < 0.01; Figures 3B,C). Furthermore, the soma size of microglia from the lesioned striatum was significantly larger than those from the intact striatum (*F*_1_,_30_ (10, *p* < 0.01; Figure 3D) with Tukey *post-hoc* testing detecting these increases to be specific to L-DOPA-treated rats (*p* < 0.05). Clear enlargements of microglial somas within the dorsal lesioned striatum can be seen in the inserts of Figure 3B. No significant interaction of variables was identified. When expressed as percentage size change in lesion versus intact striatum, there was a statistically significant difference between saline and L-DOPA groups (*t* = 2.7; df = 15; *p* < 0.05) (Figure 3E). Moreover, there was a trend toward a positive correlation between microglial soma size in the lesioned hemisphere and both AIMs scores (*r* = 0.69; *p* = 0.09) and percentage change in striatal volume (*r* = 0.76; *p* = *0.08)* for individual L-DOPA-treated rats, although neither are statistically significant (Figures 3F,G).

**FIGURE 4:**
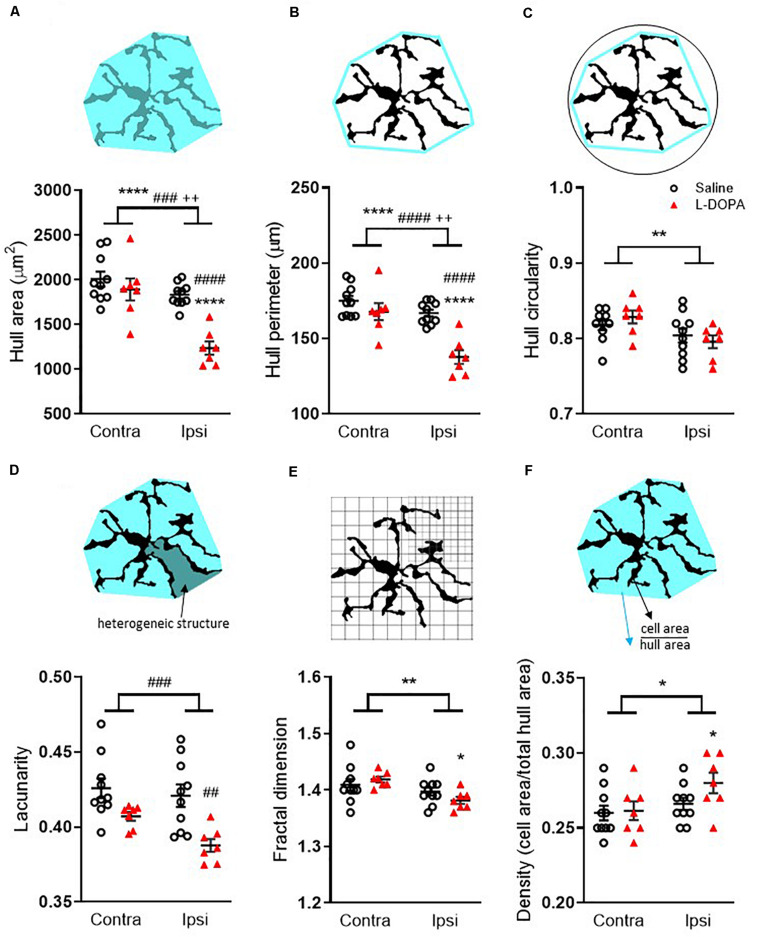
Fractal analysis of morphological changes in striatal Iba1+ microglia in L-DOPA-treated (dyskinetic) versus saline-treated (control) rats. Data are shown for **(A)** Hull area, **(B)** Hull perimeter, **(C)** Hull circularity, **(D)** Lacunarity, **(E)** Fractal dimension and **(F)** Density. The schematics above each graph illustrate the measures which were taken from 27 randomly selected microglia across the rostrocaudal extent (AP + 1.7 – AP-2.5) of the contralateral (contra) intact and ipsilateral (ipsi) lesioned striatum per rat. A full description of each parameter is given in the section “Materials and Methods.” Symbols above horizontal bars denote an overall effect of lesion (*), treatment (#) or interaction (+), while those below the bars denote *post hoc* significance versus contralateral striatum in same treatment group (*) or versus saline-treated rats in the same hemisphere (#) (two-way ANOVA with Tukey *post hoc* test). In each case 1, 2, 3 or 4 symbols represents *p* < 0.05, *p* < 0.01, *p* < 0.001 or *p* < 0.0001, respectively. Bars show mean ± SEM. Saline-treated: *n* = 10; L-DOPA -treated: *n* = 7.

Fractal analysis revealed additional morphological changes in Iba1+ microglia following L-DOPA treatment. For hull area (Figure 4A) two-way ANOVA revealed a significant effect of lesion, treatment and interaction (*F*_1_,_30_ = lesion: 26, *p* < 0.001; treatment: 19, *p* < 0.001; interaction: 8.6, *p* < 0.01). *Post hoc* analysis revealed a significant reduction in the lesioned hemisphere of L-DOPA treated rats compared to both their intact hemisphere (*p* < 0.0001) and to the lesioned hemisphere of saline-treated rats (*p* < 0.0001). For hull perimeter (Figure 4B) a similar overall effect of lesion, treatment and interaction was noted (*F*_1_,_30_ = lesion: 24, *p* < 0.001; treatment: 22, *p* < 0.001; interaction: 8.1, *p* < 0.01) accompanied by significant reductions in the lesioned hemisphere of L-DOPA treated rats compared to both their intact hemisphere (*p* < 0.0001) and to the lesioned hemisphere of saline-treated rats (*p* < 0.0001). While an overall effect of lesion to reduce hull circularity was observed (Figure 4C; (*F*_1_,_30_ = 7.6, *p* < 0.01), there was no effect of treatment. L-DOPA treatment did, however, reduce both the heterogeneity and complexity of microglia morphology. Thus, for lacunarity (Figure 4D), an overall effect of treatment was observed (*F*_1_,_30_ = 16, *p* < 0.001) which *post hoc* analysis revealed was restricted to the lesioned (ipsilateral) hemisphere (*p* < 0.01). On the other hand, an overall effect of lesion on fractal dimension was observed (*F*_1_,_30_ = 8.7, *p* < 0.01) and this was restricted to the L-DOPA treatment group (*p* < 0.05; Figure 4E). Finally, in parallel with the increase in soma size noted above, an effect of lesion was noted on individual cell density (*F*_1_,_30_ = 5, *p* < 0.05) which was found to be specifically increased in the L-DOPA treatment group (*p* < 0.05; Figure 4F). None of these fractal analysis parameters in the lesioned hemisphere were correlated with either AIMs scores or percentage change in striatal volume for individual L-DOPA-treated rats (Supplementary File 1B).

### Endothelial Markers Are Unaltered Within the 6-OHDA Lesioned Rat Striatum Following L-DOPA Treatment

**FIGURE 5:**
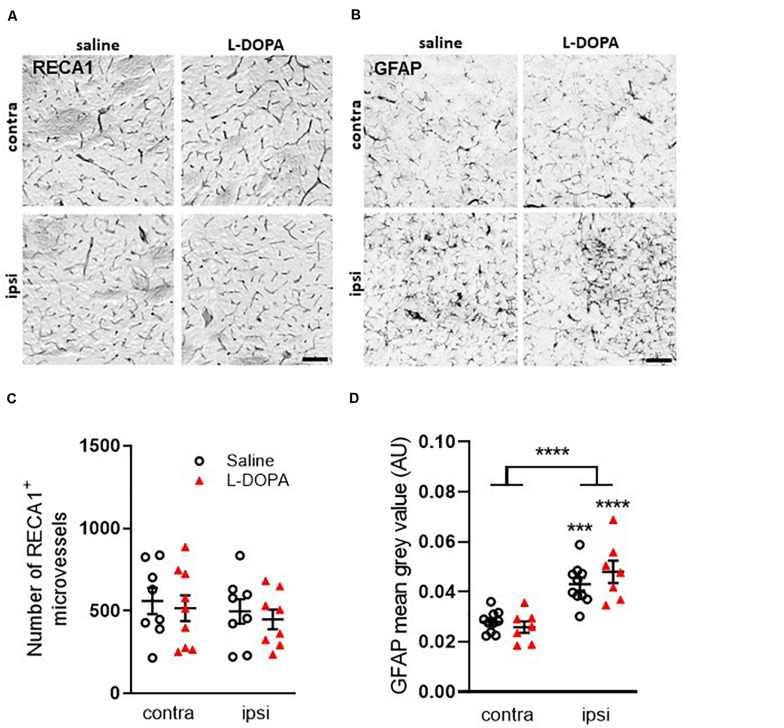
Assessment of striatal vasculature and astrocytic alterations in L-DOPA-treated (dyskinetic) versus saline-treated (control) rats. **(A,C)** Representative photomicrographs of RECA1+ vasculature or GFAP+ astrocytes within the contralateral (contra) intact and ipsilateral (ipsi) lesioned striatum of saline- and L-DOPA-treated rats. Scale bar = 100 μm. Images represent striatal region as defined in Figure 3A. **(B)** Number of RECA1+ microvessels and **(D)** Mean gray value quantification of GFAP density within the contralateral and ipsilateral striatum. **** above horizontal bar denotes an overall effect of lesion (*p* < 0.0001) while *** and **** below bar denotes *p* < 0.001 and *p* < 0.0001, respectively, versus contralateral striatum in same treatment group (two-way ANOVA with Tukey *post hoc*). Bars show mean ± SEM. Saline-treated: *n* = 10; L-DOPA-treated: *n* = 7. AU = arbitrary units.

Alterations in striatal endothelium and blood-brain barrier (BBB) have been previously identified in dyskinetic animals (Westin et al., 2006; Lindgren et al., 2009). Hence, we used RECA1 staining (a marker of endothelial cells) to investigate angiogenesis in the striatum. The number of RECA1+ microvessels was found to be highly variable within groups and no statistically significant differences emerged when comparing between the L-DOPA and saline treatment groups (*F*_1_,_30_ = 0.33, *p* > 0.05; Figures 5A,B). Similarly, the vasculature was not affected by 6-OHDA lesioning (*F*_1_,_30_ = 0.69, *p* > 0.05; Figure 5B). In line with these findings, there was no correlation between number of RECA1+ microvessels in the lesioned hemisphere and either their AIMs score (*r* = 0.017; *p* = 0.97) or percentage change in striatal volume (*r* = 0.016; *p* = *0.97)* for individual L-DOPA-treated rats.

### Striatal Astrogliosis Occurs Following 6-OHDA Lesioning but Is Unaffected by L-DOPA Treatment

Recent studies also implicate striatal astrogliosis in the development of AIMs (Bortolanza et al., 2015; Pisanu et al., 2018) therefore we analyzed striatal GFAP density. A significant increase in GFAP+ mean grey value (MGV) was observed in the lesioned hemisphere of rats within both the saline and L-DOPA treatment groups (*F*_1_,_30_ = 47, *p* < 0.0001; Tukey *post hoc*, L-DOPA treatment = *p* < 0.0001; saline treatment *p* < 0.001; Figures 5C,D). However, GFAP MGV was not affected by L-DOPA administration *per se* (*F*_1_,_30_ = 0.29, *p* > 0.05; Figures 5C,D). In line with these findings, there was no correlation between GFAP MGV in the lesioned hemisphere and either AIMs scores (*r* = 0.02; *p* = 0.96) or the percentage change in striatal volume (*r* = < 0.01; *p* = 0.98) for individual L-DOPA-treated rats.

## Discussion

The main findings of this study are that chronic L-DOPA treatment of 6-OHDA lesioned rats is associated with an apparent increase in the volume of the ipsilateral, lesioned striatum, as measured from *ex vivo* MR images, leading to a leftward shift in striatal volume asymmetry index. These macroscale changes were positively correlated with the AIMs scores of the severely dyskinetic L-DOPA treated rats, suggesting a potential link between the two. At the cellular level, neither 6-OHDA lesioning nor L-DOPA treatment affected RECA1 immunoreactivity in the striatum. While we found evidence for lesion-related increases in GFAP+ astrocyte immunoreactivity and Iba1+ microglia density, these again were unrelated to L-DOPA treatment under the current experimental conditions. Consistent with this, none of these cellular parameters were correlated with either the striatal volume expansion or AIMs scores of the L-DOPA treated rats. In contrast, chronic L-DOPA treatment was associated with an increase in Iba1+ microglia soma size specifically in the ipsilateral, lesioned striatum when compared to saline-treated rats. These changes were positively related to the striatal volume expansion and AIMs scores of individual L-DOPA treated rats, although these failed to reach statistical significance, due to the small sample size. Moreover, there were significant alterations in some morphological features of Iba1+ microglia in the lesioned striatum of L-DOPA treated rats that manifest as a less complex structure and reduced hull area and perimeter of these cells, though again these parameters were not correlated with either AIMs or MRI. Collectively, the results of the present study do not allow us to conclude whether these volume and cellular changes are causally linked to the development of dyskinesia *per se*, which requires an additional group of L-DOPA treated rats that do not show dyskinesia, as indexed by AIMs. Rather, they provide for the first time, a nexus of causality that L-DOPA treatment is associated with changes in striatal volume, detectable by MRI under conditions of dopamine depletion induced by 6-OHDA lesioning. Furthermore, this volume expansion is correlated with AIMs, a behavioral proxy for L-DOPA-induced dyskinesia. Whilst microglia appear to be involved in this process, the underlying cellular correlates of the striatal volume changes remain to be definitively established.

The potential for L-DOPA to cause gray matter volume changes as suggested by our preliminary MRI data is consistent with some observations from structural MRI studies in dyskinetic PD patients. Specifically, region-specific increases in both gray matter volume and cortical thickness are reported in dyskinetic relative to non-dyskinetic patients, although striatal volume changes were not reported in these studies (Cerasa et al., 2011, 2013a,b). Hence, whether MRI detectable volume changes occur in other rat brain regions, particularly the cortex, in response to chronic L-DOPA treatment therefore remains to be tested. This could be easily addressed using voxel-wise analysis methods such as tensor-based morphometry, which we have previously shown to be sensitive to drug-induced anatomical changes in the brain of rats bearing lesions of the nigrostriatal pathway (Vernon et al., 2014; Harrison et al., 2015). Such analyses, however, require larger sample sizes and higher quality MR images than were available here; hence this remains an important goal for future studies. Whether our findings with regard to striatal volume changes generalize to other experimental *in vivo* models of relevance for PD following L-DOPA treatment also needs to be confirmed. Nevertheless, our data provide the first evidence for a shift in ipsilateral striatal volume following chronic L-DOPA treatment at a dose that induced dyskinesia and find a positive correlation between these phenomena, plausibly suggesting they are linked. Our sample size was, however, small and our study was only cross-sectional in nature. As already stated, however, whether the striatal volume change is a cause or consequence of dyskinesia following chronic L-DOPA treatment remains to be established. Longitudinal *in vivo* multi-modal MRI studies, based on our initial positive findings herein, are therefore also an important future research goal to confirm and extend these *ex vivo* findings. Importantly, the current data provide the necessary effect sizes to ensure such as study is adequately powered. Future studies should also reduce the interval between last L-DOPA dose and MRI analysis such that any potential impact of chronic L-DOPA-related changes subsiding can be minimized.

A secondary objective of our study was to establish plausible cellular correlates of the change in striatal volume following chronic L-DOPA treatment in 6-OHDA lesioned rats. Previous studies have reported astrogliosis in the denervated dyskinetic striatum (Bortolanza et al., 2015) and such an effect may underlie an increase in volume, as noted previously for the hippocampus (Biedermann et al., 2016). However, while we observed an increase in GFAP immunoreactivity, reflecting astrogliosis, within the ipsilateral lesioned striatum, there were no group-wise differences between vehicle and L-DOPA treated rats, nor any correlation between GFAP signal and either AIMs or striatal volume change, arguing against a major contribution of astrogliosis to the volume expansion noted here. These data are at odds with some previous studies which, like ours, showed elevated GFAP in the lesioned striatum but, in contrast, showed a further elevation after L-DOPA treatment (Bortolanza et al., 2015; Ramírez-García et al., 2015). These discrepancies may reflect the alternative route or higher doses of L-DOPA administered in these previous studies (30 and 100 mg/kg orally, respectively) when compared to 6.25 mg/kg s.c. used here. Nevertheless, our data do not support increased GFAP+ astrocyte density as a contributing factor to the volume expansion seen in rats with following chronic L-DOPA dosing.

There is also evidence suggesting L-DOPA exposure modifies microvasculature within the brain. Studies have reported L-DOPA treated dyskinetic rats to have enhanced blood brain barrier (BBB) permeability, increased cerebral blood flow and increased blood vessel length, supportive of angiogenesis (Westin et al., 2006; Lindgren et al., 2009; Ohlin et al., 2012; Boi et al., 2019). We found no evidence, however, for any treatment-related effect on RECA1 immunoreactivity, neither was RECA1 immunoreactivity correlated with AIMs or striatal volume change. In line with this view, studies of the striatal microvasculature in rat LID models have focussed on either endothelial proliferation or markers of immature endothelium, which would be consistent with angiogenesis. For example, Westin et al. (2006) did not find statistically significant differences in blood vessel length in the striatum, although such changes were present in the substantia nigra pars reticulata and entopeduncular nucleus. That said, profound increases in striatal cerebral blood flow are reported in rat LID models (Ohlin et al., 2012; Lerner et al., 2016). Hence, further studies are therefore required to confirm whether increased vascularisation contributes to the striatal volume expansion seen in the dyskinetic state, including, for example, morphological analysis of cerebral blood vessels. Extending such analysis to discrete striatal sub-regions, for example, the motor regions may also yield informative data.

Consistent with previous studies, we observed an increase in Iba1+ microglia density in the ipsilateral, lesioned striatum that was not further elevated in L-DOPA-treated animals (Fang et al., 2011). Others have reported further elevations in L-DOPA-treated animals when employing OX-42 as the microglial marker (Bortolanza et al., 2015; Pisanu et al., 2018; Boi et al., 2019). Since OX-42 also labels neutrophils, which Iba1 does not (Ji et al., 2007; Jeong et al., 2013), the discrepancies here may reflect an underlying contribution from neutrophils in this model, which remains to be explored. Regardless, the ability of pharmacological agents that arrest the OX-42-mediated neuroimmune response to reverse LID provides strong evidence in favor of the link between neuroinflammation and LID (Bortolanza et al., 2015; Pisanu et al., 2018; Boi et al., 2019). Although we did not observe an increase in Iba1+ cell density related to L-DOPA treatment, we did find a selective increase in Iba1+ microglia soma size in the ipsilateral striatum of L-DOPA treated rats. Although this was positively related to striatal volume change and AIMs scores for individual rats in the L-DOPA treatment group, these correlations failed to reach statistical significance (*p* = 0.08 for volume change; *p* = 0.09 for AIMs, respectively). Power calculations performed using “Exact, correlation bivariate normal model” in G^∗^power (2-tailed; power = 0.95, alpha = 0.05; Faul et al., 2009) revealed that group sizes of *n* = 10 per group for AIMS (r squared = 0.48) and *n* = 8 per group for volume change (*r* squared = 0.58) were required to achieve *p* < 0.05. Our study was not, however, powered to determine links between microglial changes and both the striatal volume change and AIMs, which may now be explored in future studies, informed by these sample size calculations.

A more detailed, analysis of the morphological features of Iba1+ microglia, revealed significant reductions in hull area and perimeter in the lesioned striatum which related to L-DOPA treatment. Coupled with L-DOPA related reductions in lacunarity and fractal dimension, these changes infer a less heterogenous, less branched morphology being adopted, perhaps indicating a more activated phenotype of microglia under these experimental conditions. However, once again, these parameters were not correlated with either AIMs or MRI in individual L-DOPA-treated, 6-OHDA-lesioned rats, so cannot explain these phenomena.

Whilst these data add to the growing link between chronic L-DOPA treatment and microgliosis, they leave open the question of what other accompanying cellular changes may be related to the L-DOPA mediated increase in ipsilateral striatal volume seen here. Addressing this point directly, there is mounting evidence that the development of LID is associated with maladaptive synaptic plasticity in striatal medium spiny and cortical pyramidal neurons that is accompanied by morphological changes (Deutch et al., 2007; Zhang et al., 2013; Fieblinger et al., 2014; Nishijima et al., 2014; Suarez et al., 2014, 2016; Ueno et al., 2014, 2017; Gomez et al., 2019). Several studies have found evidence for significantly diminished density of dendritic spines on both D1-receptor (D1R) and D2-receptor (D2R) expressing medium spiny neurons (MSNs) in the rodent striatum following dopamine depletion, which are not associated with a decrease in MSN number, density, or cell soma size (Suarez et al., 2014, 2016; Gomez et al., 2019). Others report a similar reduction in spine density of D2R expressing MSNs, but not D1R expressing MSNs (Fieblinger et al., 2014; Nishijima et al., 2014). These data are consistent with reports of decreased striatal dendritic spine density in *post-mortem* brain tissue from PD cases as compared to controls (Stephens et al., 2005). They are also consistent with the reduced ipsilateral striatal volume noted here in dopamine-depleted rats treated with saline and in previous MRI studies in rat and primate models of PD (Vernon et al., 2010, 2011; Westphal et al., 2016; Modo et al., 2017). Of particular relevance here, chronic L-DOPA treatment, at doses that induce dyskinesia, restores spine density on D2R expressing MSNs (Fieblinger et al., 2014; Nishijima et al., 2014; Suarez et al., 2014, 2016; Gomez et al., 2019) and, while not restoring spine density, leads to increases spine size in D1R expressing MSNs (Fieblinger et al., 2014; Nishijima et al., 2014). Chronic L-DOPA treatment in dopamine-depleted rats also leads to a marked expansion of the dendritic arbor in a large proportion of striatonigral neurons (Fieblinger et al., 2018). Sprouting of serotonergic axons that form increased synaptic contacts is also reported in the striatum of dopamine depleted rats following chronic dosing with doses of L-DOPA that induce dyskinesia (Rylander et al., 2010). Changes in dendritic spine density are also suggested to explain the largest proportion of the variance in gray matter volume changes in the mouse brain, in the context of learning-induced structural changes (Keifer et al., 2015). Hence, it is plausible to suggest that the apparent volume increases following chronic L-DOPA treatment observed herein may reflect changes in spine density in D2R expressing MSNs and spine size in D1R expressing MSNs. Testing this hypothesis directly will be an important goal for future studies. Nonetheless, these data are also potentially important for the interpretation of our microglia findings. Specifically, there is an increasing body of evidence that microglia-neuron interactions are important in modulating the density of dendritic spines (Paolicelli et al., 2011). In the current study, however, the fixation of the brain tissue for *ex vivo* MR imaging precluded an analysis of dendritic spine density, which typically requires fresh frozen tissue combined with Golgi-cox impregnation. Further studies are therefore necessary to explore whether changes in striatal microglia morphology following L-DOPA treatment are related to changes in dendritic spine density.

Accepting that L-DOPA treatment may modulate gray matter volume, our findings may also have wider implications with regard to the interpretation of structural MRI changes in PD patients, which could be a consequence of disease, of treatment or an interaction between the two. It should be noted, however, that longitudinal MRI studies of non-dyskinetic PD patients have reported no correlation between structural brain changes and L-DOPA daily equivalents, although these patients were not dyskinetic at the time of scanning (Mak et al., 2015, 2017). Furthermore, two important limitations of our study in this respect is the absence of sham-lesion control group and L-DOPA treated rats that do not show dyskinesia, as indexed by AIMs. As such, although we have previously demonstrated across multiple neurotoxin models that dopamine depletion is associated with a reduction in striatal volume relative to sham-operated controls (Vernon et al., 2010, 2011; Westphal et al., 2016; Modo et al., 2017), whether such volume changes are reversed by L-DOPA treatment cannot be addressed by the current study and should be investigated further before making any inference in this regard, ideally using *in vivo* imaging to control for potential contributions of post-mortem tissue handling in the effects observed.

## Conclusion

Collectively, these preliminary data suggest chronic L-DOPA treatment in 6-OHDA lesioned rats is associated with an apparent increase in volume of the ipsilateral lesioned striatum, which is also associated with the development of AIMs. These changes were accompanied by underlying microgliosis, although these did not account directly for the volume expansion, which may further reflect other previously established alterations in synaptic morphology. Based on our initial findings, however, longitudinal multi-modal MRI-histology studies are clearly warranted to establish the effects of chronic L-DOPA treatment and dyskinesia across the brain.

## Data Availability Statement

The raw data supporting the conclusions of this article will be made available by the authors, without undue reservation.

## Ethics Statement

The animal study was reviewed and approved by King’s College London Animal Welfare and Ethical Review Body, King’s College London, Guy’s Campus, London SE1 1UL United Kingdom.

## Author Contributions

CF performed the dyskinesia induction and assessments. AAL and AV performed the MR image analysis with support from WC. EF performed all the post-mortem immunohistochemistry. AV and EF performed the statistical analyses. AV and SD planned and generated funding for the studies. AV, EF, and SD wrote the manuscript. All authors contributed to manuscript revision, read and approved the submitted version.

## Conflict of Interest

The authors declare that the research was conducted in the absence of any commercial or financial relationships that could be construed as a potential conflict of interest.
