# Supplementary figures and images for: Neuroanatomical and Microglial Alterations in the Striatum of Levodopa-Treated, Dyskinetic Hemi-Parkinsonian Rats
Source: Front Neurosci. 2020 Sep 15;14:567222. doi: 10.3389/fnins.2020.567222 (PMC7522511; doi:10.3389/fnins.2020.567222)

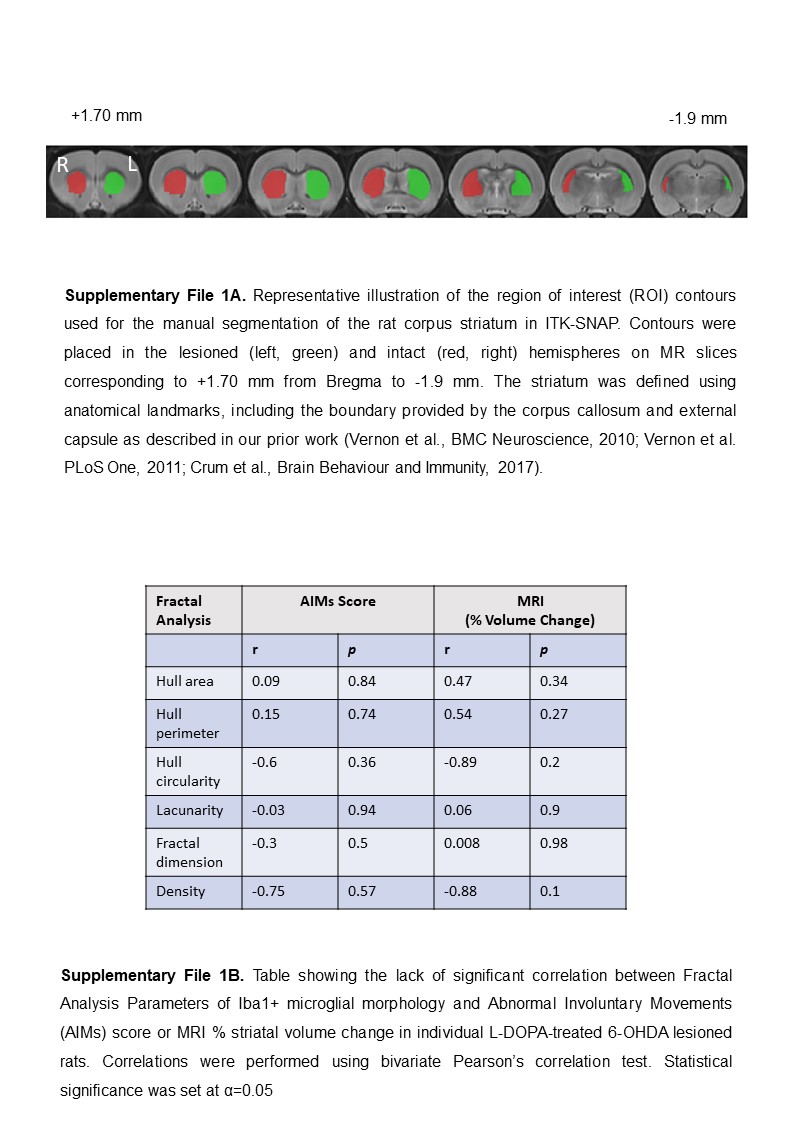

Supplement: Supplementary file 1 [file Image_1.JPEG]
